# Patient reported measures of continuity of care and health outcomes: a systematic review

**DOI:** 10.1186/s12875-024-02545-8

**Published:** 2024-08-19

**Authors:** Patrick Burch, Alex Walter, Stuart Stewart, Peter Bower

**Affiliations:** Centre for Primary Care and Health Services Research, Institute of Population Health, University of Manchester, Manchester, England

## Abstract

**Background:**

There is a considerable amount of research showing an association between continuity of care and improved health outcomes. However, the methods used in most studies examine only the pattern of interactions between patients and clinicians through administrative measures of continuity. The patient experience of continuity can also be measured by using patient reported experience measures. Unlike administrative measures, these can allow elements of continuity such as the presence of information or how joined up care is between providers to be measured. Patient experienced continuity is a marker of healthcare quality in its own right. However, it is unclear if, like administrative measures, patient reported continuity is also linked to positive health outcomes.

**Methods:**

Cohort and interventional studies that examined the relationship between patient reported continuity of care and a health outcome were eligible for inclusion. Medline, EMBASE, CINAHL and the Cochrane Library were searched in April 2021. Citation searching of published continuity measures was also performed. QUIP and Cochrane risk of bias tools were used to assess study quality. A box-score method was used for study synthesis.

**Results:**

Nineteen studies were eligible for inclusion. 15 studies measured continuity using a validated, multifactorial questionnaire or the continuity/co-ordination subscale of another instrument. Two studies placed patients into discrete groups of continuity based on pre-defined questions, one used a bespoke questionnaire, one calculated an administrative measure of continuity using patient reported data. Outcome measures examined were quality of life (*n* = 11), self-reported health status (*n* = 8), emergency department use or hospitalisation (*n* = 7), indicators of function or wellbeing (*n* = 6), mortality (*n* = 4) and physiological measures (*n* = 2). Analysis was limited by the relatively small number of hetrogenous studies. The majority of studies showed a link between at least one measure of continuity and one health outcome.

**Conclusion:**

Whilst there is emerging evidence of a link between patient reported continuity and several outcomes, the evidence is not as strong as that for administrative measures of continuity. This may be because administrative measures record something different to patient reported measures, or that studies using patient reported measures are smaller and less able to detect smaller effects. Future research should use larger sample sizes to clarify if a link does exist and what the potential mechanisms underlying such a link could be. When measuring continuity, researchers and health system administrators should carefully consider what type of continuity measure is most appropriate.

**Supplementary Information:**

The online version contains supplementary material available at 10.1186/s12875-024-02545-8.

## Introduction

Continuity of primary care is associated with multiple positive outcomes including reduced hospitals admissions, lower costs and a reduction in mortality [1–3]. Providing continuity is often seen as opposed to providing rapid access to appointments [4] and many health systems have chosen to focus primary care policy on access rather than continuity [5–7]. Continuity has fallen in several primary care systems and this has led to calls to improve it [8, 9]. However, it is sometimes unclear exactly what continuity is and what should be improved.

In its most basic form, continuity of care can be defined as a continuous relationship between a patient and a healthcare professional [10]. However, from the patient perspective, continuity of care can also be experienced as joined up seamless care from multiple providers [11].

One of the most commonly cited models of continuity by Haggerty et al. defines continuity as

“*…the degree to which a series of discrete healthcare events is experienced as coherent and connected and consistent with the patient’s medical needs and personal context. Continuity of care is distinguished from other attributes of care by two core elements—care over time and the focus on individual patients”* [11].

It then breaks continuity down into three parts (see Table 1) [11]. Other academic models of patient continuity exists but they contain elements which are broadly analogous [10, 12–14].

**Table 1:** Types of continuity

• Relational continuity – An ongoing relationship between the patient and one (or more than one) provider of healthcare. This is closely related to the concept of longitudinal continuity which is commonly used as a proxy marker of relational continuity [15].
• Informational continuity – Clinicians and patients having appropriate access to information to enable healthcare
• Management continuity – the extent to which the approach to healthcare over time, and potentially between different providers, is responsive, joined up and coherent.

Continuity can be measured through administrative measures or by asking patients about their experience of continuity [16]. Administrative mesures are commonly used as they allow continuity to be calculated easily for large numbers of patient consultations. Administraive measures capture one element of continuity – the frequency or pattern of professionals seen by a patient [16, 17]. There are multiple studies and several systematic reviews showing that better health outcomes are associated with administrative measures of continuity of care [1, 2, 18, 19]. One of the most recent of these reviews used a box-score method to assess the relationship between reduced mortality and continuity (i.e., counting the numbers of studies reporting significant and non-significant relationships) [18]. The review examined thirteen studies and found a positive association in nine. Administrative measures of continuity cannot capture aspects of continuity such as informational or management continuity or the nature of the relationship between the patient and clinicians. To address this, there have been several patient-reported experience measures (PREMs) of continuity developed that attempt to capture the patient experience of continuity beyond the pattern in which they see particular clinicians [14, 17, 20, 21]. Studies have shown a variable correlation between administrative and patient reported measures of continity and their relationship to health outcomes [22]. Pearson correlation co-efficients vary between 0.11 and 0.87 depending on what is measured and how [23, 24]. This suggests that they are capturing different things and that both measures have their uses and drawbacks [23, 25]. Patients may have good administrative measures of continuity but report a poor experience. Conversely, administrative measures of continuity may be poor, but a patient may report a high level of experienced continuity. Patient experienced continuity and patient satisfaction with healthcare is an aim in its own right in many healthcare systems [26]. Whilst this is laudable, it may be unclear to policy makers if prioritising patient-experienced continuity will improve health outcomes.

This review seeks to answer two questions.

1. Is patient reported continuity of care associated with positive health outcomes?
2. Are particular types of patient reported continuity (relational, informational or management) associated with positive health outcomes?

## Methods

A review protocol was registered with PROSPERO in June 2021 (ID: CRD42021246606).

### Search strategy

A structured search was undertaken using appropriate search terms on Medline, EMBASE, CINAHL and the Cochrane Library in April 2021 (see Appendix). The searches were limited to the last 20 years. This age limitation reflects the period in which the more holistic description of continuity (as exemplified by Haggerty et al. 2003) became more prominent. In addition to database searches, existing reviews of PREMs of continuity and co-ordination were searched for appropriate measures. Citation searching of these measures was then undertaken to locate studies that used these outcome measures.

### Inclusion criteria

Full text papers were reviewed if the title or abstract suggested that the paper measured (a) continuity through a PREM and (b) a health outcome. Health outcomes were defined as outcomes that measured a direct effect on patient health (e.g., health status) or patient use of emergency or inpatient care. Papers with outcomes relating to patient satisfaction or satisfaction with a particular service were excluded as were process measures (such as quality of documentation, cost to health care provider). Cohort and interventional studies were eligible for inclusion, if they reported data on the relationship between continuity and a relevant health outcome. Cross-sectional studies were excluded because of the risk of recall bias [27].

The majority of participants in a study had to be aged over 16, based in a healthcare setting and receiving healthcare from healthcare professionals (medical or non-medical). We felt that patients under 16 were unlikely to be asked to fill out continuity PREMs. Studies that used PREMs to quantitatively measure one or more elements of experienced continuity of care or coordination were eligible for inclusion [11]. Any PREMs that could map to one or more of the three key elements of Haggerty’s definition (Table 1) definition were eligible for inclusion. The types of continuity measured by each study were mapped to the Haggerty concepts of continuity by at least two reviewers independently. Our search also included patient reported measures of co-ordination, as a previous review of continuity PREMs highlighted the conceptual overlap between patient experienced continuity and some measures of patient experienced co-ordination [17]. Whilst there are different definitions of co-ordination, the concept of patient perceived co-ordination is arguably the same as management continuity [13, 14, 28]. Patient reported measures of care co-ordination were reviewed by two reviewers to see whether they measured the concept of management continuity. Because of the overlap between concepts of continuity and other theories (e.g., patient-centred care, quality of care), in studies where it was not clear that continuity was being measured, agreement, with documented reasons, was made about their inclusion/exclusion after discussion between three of the reviewers (PB, SS and AW). Disagreements were resolved by documented group discussion. Some PREMs measured concepts of continuity alongside other concepts such as access. These studies were eligible for inclusion only if measurements of continuity were reported and analysed separately.

### Data abstraction

All titles/abstracts were initially screened by one reviewer (PB). 20% of the abstracts were independently reviewed by 2 other reviewers (SS and AW), blinded to the results of the initial screening. All full text reviews were done by two blinded reviewers independently. Disagreements were resolved by group discussion between PB, SS, AW and PBo. Excel was used for collation of search results, titles, and abstracts. Rayyan was used in the full text review process.

Data extraction was performed independently by two reviewers. The following data were extracted to an Excel spreadsheet: study design, setting, participant inclusion criteria, method of measurement of continuity, type of continuity measured, outcomes analysed, temporal relationship of continuity to outcomes in the study, co-variates, and quantitative data for continuity measures and outcomes. Disagreements were resolved by documented discussion or involvement of a third reviewer.

### Study risk of bias assessment

Cohort studies were assessed for risk of bias at a study level using the QUIP tool by two reviewers acting independently [29]. Trials were assessed using the Cochrane risk of bias tool. The use of the QUIP tool was a deviation from the review protocol as the Ottowa-Newcastle tool in the protocol was less suitable for use on the type of cohort studies returned in the search. Any disagreements in rating were resolved by documented discussion.

### Analysis

As outlined in our original protocol, our preferred analysis strategy was to perform meta-analysis. However, we were unable to do this as insufficient numbers of studies reported data amenable to the calculation of an effect size. Instead, we used a box-score method [30]. This involved assessing and tabulating the relationship between each continuity measure and each outcome in each study. These relationships were recorded as either positive, negative or non-significant (using a conventional *p* value of < 0.05 as our cut off for significance). Advantages and disadvantages of this method are explored in the discussion section. Where a study used both bivariate analysis and multivariate analysis, the results from the multivariate analysis were extracted. Results were marked as “mixed” where more than one measure for an outcome was used and the significance/direction differed between outcome measures. Sensitivity analysis of study quality and size was carried out.

## Results

**Fig. 1:**
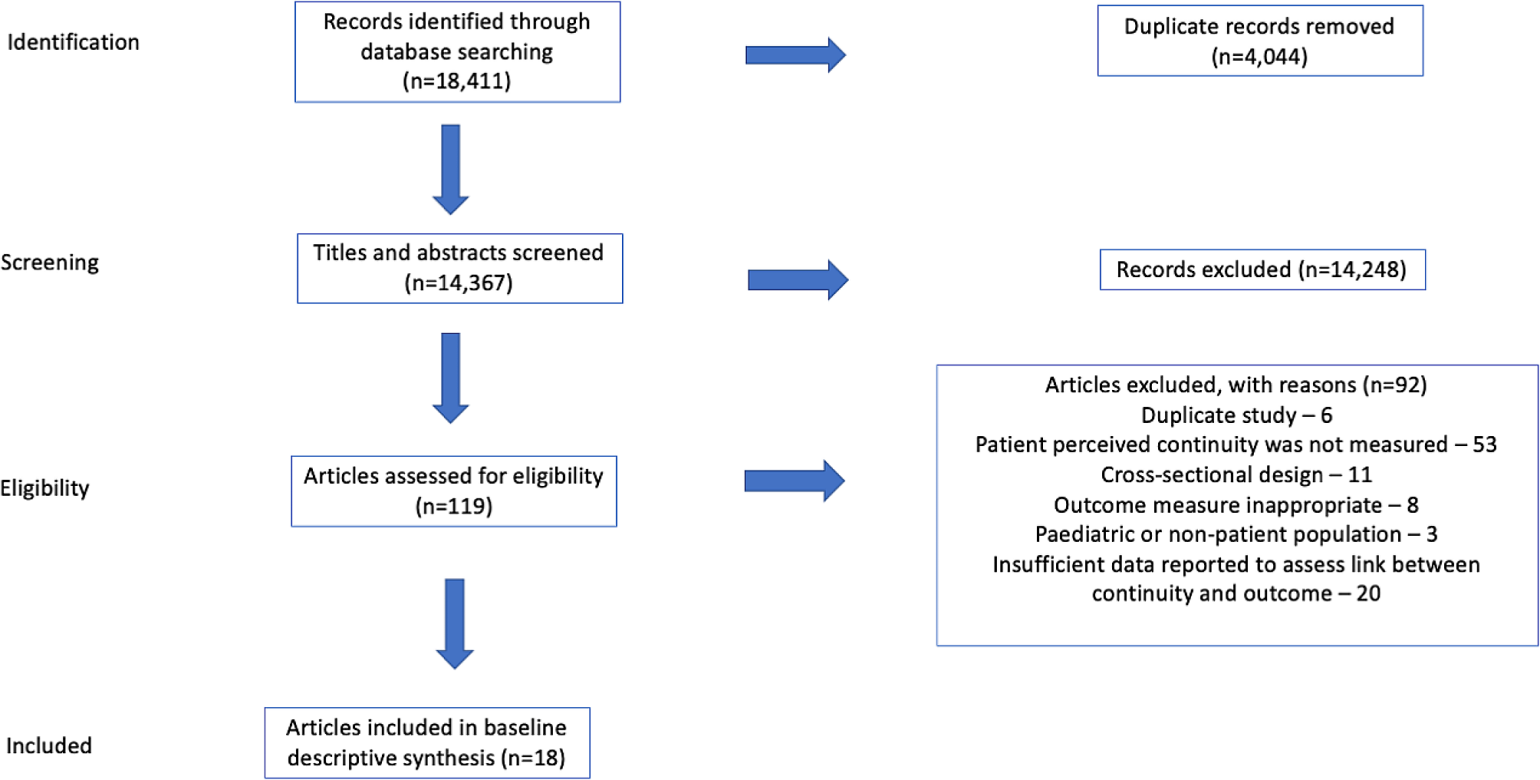
Results of search strategy –NB. 18 studies provided 19 assessments

Figure 1 shows the search results and number of inclusions/exclusions. Studies were excluded for a number of reasons including; having inappropriate outcome measures [31], focusing on non-adult patient populations [32] and reporting insufficient data to examine the relationship between continuity and outcomes [33]. All studies are described in Table 2.

**Table 2:** Data extraction table

Study number	Author, reference	Patient group, setting	Design(A) and number of patients	Continuity measure(B)	Types of continuity measured and reported(C)	Outcomes measured and reported(D)	Relationship between continuity measure and outco€(E)	Type of analysis (F)	Risk of bias rating	Results summary(G)
1	Adair [19]	Adult patients with a diagnosis of severe mental illness, Canada	C 486	1	Ov	QoL, PRHS	E	M, S	Low to moderate	QoL +, PRHS n
2a	Beesley [20]	Patients with pancreatic cancer who had curative surgery, Australia	C 54	1	In, M	QoL, PRHS, mortality	M	M	Moderate	QoL +, PRHS +, mortality n
2b	Beesley [20]	Patients with pancreatic cancer who did not receive curative surgery, Australia	C 58	1	In, M	QoL, PRHS, mortality	M	M	Moderate	QoL n, PRHS n, mortality n
3	Bentler [13]	Medicare beneficiaries aged 65+, USA	C 1219	2	R	ED, mortality	B	M	Moderate	ED+, Mortality+
4	Catty [21]	Patients with non psychotic mental illness using community mental health teams aged 18–65, UK	C 98	1	R, M	QoL, ED, PRHS, empowerment	X	M	Moderate	QoL M, ED n PRHS n, empowerment n
5	Catty [22]	Patients with psychotic mental illness using community mental health teams aged 18–65, UK	C 180	1	R, M	QoL, ED PRHS	X	M	Moderate	QoL Mn ED n, PRHS n
6	Cohen Castel [23]	Patients with cancer who were receiving their first prescription of oral chemotherapy agents, Israel	C 150	1	R, M	QoL, medication adherence	B	M, S	Moderate	QoL n, medication adherence M+
7	Gulliford [24]	Patients with type 2 diabetes, UK	C 209	1	Ov	QoL, HbA1c, blood pressure, weight, BMI	M	M	Moderate	QoL n, HbA1c n, blood pressure n, weight n, BMI n
8	Hudon [25]	(2 cohorts) Members of the general population and Primary care patients aged 25–75, Canada	C 1769	2	M	ED	M	M	Moderate to high	ED+
9	Humphries [26]	Patients admitted to hospital with a non-communicable disease, India	C 546	4	In	ED, mortality, disease deterioration	M	M	Moderate	ED n, Mortality n, disease deterioration +
10	Hustoft [27]	Patients accepted for rehabilitation in a rehabilitation centre, Norway	C 984	1	R, M	PRHS, disability	E	M	Moderate	PRHS +, Disability n
11	Kaneko [28]	Members of the general population aged 65 and over, Japan	C 740	2	R, M	ED	B	M	Moderate	ED -M
12	Kim [29]	Subjects with alcohol, cocaine, and/or heroin use disorders who initiated primary care after being discharged from a detoxification program, USA	C 183	2	R	The presence of any substance use, Addiction severity	M	M	Moderate	Substance use n, addiction severity n
13	King [30]	Cancer patients who attended hospital care with breast, lung or colorectal cancer, UK	C 206	1	Ov	QoL, pRHs, Care needs	M	M	Low to moderate	QoL n, PRHS n, care needs -
14	Konrad [31]	White or black patients aged over 65 with white or black primary care physicians, USA	C 4136	4	R	Severity of hypertension, presence of undetected hypertension, undetected severe hypertension, ever being told of high blood pressure, taking blood pressure medication	B	M	High	Severity of hypertension n, presence of undetected hypertension +, undetected severe hypertension +, ever being hold of high blood pressure -, taking blood pressure medication -
15	Mold [32]	Patients aged over 65 attending a primary care physician, USA	C 854	2	R, M	QoL, Mortality	B	M	Low to moderate	QoL n, Mortality n
16	Uijen [33]	Patients with COPD aged over 35, Netherlands	T 180	3	M	QoL	M	U	High	Qol*
17	Valaker [34]	Patients who underwent percutaneous coronary intervention (PCI), Norway	C 1695	1	Ov, R,In, M	Qol, PRHS	X	M, S	Low to moderate	Qol+, PRHS nM
18	Van Walraven [35]	Adult patients discharged after admission to hospital, USA	C 3876	5	R	ED, Mortality	M	M	Moderate to high	ED +, Mortality n

A: C = cohort T = trial

B: 1 = Validated multifactorial measure of continuity 2 = Continuity/co-ordination subscale from another validated instrument 3 = Bespoke questionnaire 4 = simple groupings 5 = Patient recalled Usual Provider of Care (UPC) index

C: Ov = Overall, R = Relational/longitudinal, In = Informational, M = Management/co-ordination,

D: QoL = Quality of life, PRHS = Patient reported health status, ED = Emergency department use or hospitalisation. Other measures are name in full

E: M = Continuity measured more than once during study – outcomes at end, X = Continuity and outcomes measured more than once during study B = Continuity measured before outcomes, E = Continuity examined at end of study along with outcomes, D = Outcomes examined before and after measurement of continuity

F: M = Analysis adjusted for multiple confounders S = Bivariate(unadjusted) analysis U = unclear N.B. studies may have used different analyses methods for different outcomes/continuity types

G: +=positive *=positive but insufficient data to establish if statistically significant. *n* = no significant association -=negative association. M = Mixed results. N.B As some studies measure more than one type of continuity, there may be more than one result for a particular outcome. Studies that use three of more measures of a particular outcome are reported as–+ or - if a majority of outcome measures are–+ or -

### Study settings

Studies took place in 9 different, mostly economically developed, countries. Studies were set in primary care [5], hospital/specialist outpatient [7], hospital in-patient [5], or the general population [2].

### Study design and assessment of bias

All included studies, apart from one trial [34], were cohort studies. Study duration varied from 2 months to 5 years. Most studies were rated as being low-moderate or moderate risk of bias, due to outcomes being patient reported, issues with recruitment, inadequately describing cohort populations, significant rates of attrition and/or failure to account for patients lost to follow up.

### Measurement of continuity

The majority of the studies (15/19) measured continuity using a validated, multifactorial patient reported measure of continuity or using the continuity/co-ordination subscale of another validated instrument. Two studies placed patients into discrete groups of continuity based on answers to pre-defined questions (e.g., do you have a regular GP that you see? ) [35, 36], one used a bespoke questionnaire [34], and one calculated an administrative measure of continuity (UPC – Usual Provider of Care index) using patient reported visit data collected from patient interviews [37]. Ten studies reported more than one type of patient reported continuity, four reported relational continuity, three reported overall continuity, one informational continuity and one management continuity.

### Study outcomes

Most of the studies reported more than one outcome measure. To enable comparison across studies we grouped the most common outcome measures together. These were quality of life (*n* = 11), self-reported health status (*n* = 8), emergency department use or hospitalisation (*n* = 7), and mortality (*n* = 4). Other outcomes reported included physiological parameters e.g., blood pressure or blood test parameters (*n* = 2) [36, 38] and other indicators of functioning or well-being (*n* = 6).

### Association between outcomes and continuity measures

Twelve of the nineteen studies demonstrated at least one statistically significant association between at least one patient reported measure of continuity and at least one outcome. However, ten of these studies examined more than one outcome measure. Two of these significant studies showed negative findings; better informational continuity was associated with worse self-reported disease status [35] and improved continuity was related to increased admissions and ED use [39]. Four studies demonstrated no association between measures of continuity and any health outcomes.

The four most commonly reported types of outcomes were analysed separately (Table 3). All the outcomes had a majority of studies showing no significant association with continuity or a mixed/unclear association. Sensitivity analysis of the results in Table 3, excluding high and moderate-high risk studies, did not change this finding. Each of these outcomes were also examined in relation to the type of continuity that was measured (Table 4) Apart from the relationship between informational continuity and quality or life, all other combinations of continuity type/outcome had a majority of studies showing no significant association with continuity or a mixed/unclear association. However, the relationship between informational continuity and quality of life was only examined in two separate studies [40, 41]. One of these studies contained less than 100 patients and was removed when sensitivity analysis of study size was carried out [40]. Sensitivity analysis of the results in Table 4, excluding high and moderate-high risk studies, did not change the findings.

Two sensitivity analyses were carried out (a) removing all studies with less than 100 participants and (b) those with less than 1000 participants. There were only five studies with at least 1000 participants. These all showed at least one positive association between continuity and health outcome. Of note, three of these five studies examined emergency department use/readmissions and all three found a significant positive association.

**Table 3:** Outcome measures association with continuity by study

Outcome (no. of studies)	Significant association	Mixed/uncertain	No association
Quality of life (11)	3 (studies 1, 2a, 17)	3 (studies 4, 5, 16)	5 (studies 2b, 6, 7, 13, 15)
Patient reported health status (8)	2 (studies 2a, 10)	1 (study 17)	5 (studies 1, 2b, 4, 5, 13)
Urgent admission/ED use (7)	3 (studies 13, 8, 18)	1 (study 11)	3 (studies 4, 5, 9)
Mortality (6)	1 (study 3)	0	5 (studies 2a, 2b, 9, 15, 18)

**Table 4:** Outcome by continuity type. N.B. Study numbers relate to study number in Table 2 (supplementary file)

Continuity type/Outcome	Overall	Relational	Informational	Management
Quality of life	Significant association 1 No association 7, 13	Significant association10, 17 Mixed results 4,5 No association 6, 15	Significant association 2a, 17 No association 2b	Significant association 10,17 Mixed result 16 No association4,5,6, 15
Patient reported health status	No association 1, 13, 17	Mixed results 17 No association 4,5	Significant association 2a Mixed results 17 No association 2b	Mixed results 17 No association 4,5
Urgent admission/ED use		Significant association 3, 18 Mixed results 11 No association 4,5	No association 9	Significant association 8, 11 No association 4,5
Mortality		Significant association 3 No association 15, 18	No association 9	No association 2a,2b, 15
Other outcomes	Significant association 13 No association 7	Mixed results 6, 14 No association4,5, 10, 12	Significant association 9	Significant association 6 No association 4,5, 10

## Discussion

Continuity of care is a multi-dimensional concept that is often linked to positive health outcomes. There is strong evidence that administrative measures of continuity are associated with improved health outcomes including a reduction in mortality, healthcare costs and utilisation of healthcare [3, 18, 19]. Our interpretation of the evidence in this review is that there is an emerging link between patient reported continuity and health outcomes. Most studies in the review contained at least one significant association between continuity and a health outcome. However, when outcome measures were examined individually, the findings were less consistent.

The evidence for a link between patient reported continuity is not as strong as that for administrative measures. There are several possible explanations for this. The review retrieved a relatively small number of studies that examined a range of different outcomes, in different patient populations, in different settings, using different outcomes, and different measures of continuity. This resulted in small numbers of studies examining the relationship of a particular measure of continuity with a particular outcome (Table 4). The studies in the review took place in a wide variety of country and healthcare settings and it may be that the effects of continuity vary in different contexts. Finally, in comparison to studies of administrative measures of continuity, the studies in this review were small: the median number of participants in the studies was 486, compared to 39,249 in a recent systematic review examining administrative measures of continuity [18]. Smaller studies are less able to detect small effect sizes and this may be the principle reason for the difference between the results of this review and previous reviews of administrative measures of continuity. When studies with less than 1000 participants were excluded, all remaining studies showed at least one positive finding and there was a consistent association between reduction in emergency department use/re-admissions and continuity. This suggests that a modest association between certain outcomes and patient reported continuity may be present but, due to effect size, larger studies are needed to demonstrate it. The box score method does not take account of differential size of studies.

Continuity is not a concept that is universally agreed upon. We mapped concepts of continuity onto the commonly used Haggerty framework [11]. Apart from the use of the Nijmegen Continuity of care questionnaire in three studies [42], all studies measured continuity using different methods and concepts of continuity. We could have used other theoretical constructs of continuity for the mapping of measures. It was not possible to find the exact questions asked of patients in every study. We therefore mapped several of the continuity measures based on higher level descriptions given by the authors. The diversity of patient measures may account for some of the variability in findings between studies. However, it may be that the nature of continuity captured by patient reported measures is less closely linked to health outcomes than that captured by administrative measures. Administrative measures capture the pattern of interactions between patients and clinicians. All studies in this review (apart from Study 18) use PREMs that attempt to capture something different to the pattern in which a patient sees a clinician. Depending on the specific measure used, this includes: aspects of information transfer between services, how joined up care was between different providers and the nature of the patient-clinician relationship. PREMs can only capture what the patient perceives and remembers. The experience of continuity for the patient is important in its own right. However, it may be that the aspects of continuity that are most linked to positive health outcomes are best reflected by administrative measures. Sidaway-Lee et al. have hypothesised why relational continuity may be linked to health outcomes [43]. This includes the ability for a clinician to think more holistically and the motivation to “go the extra mile” for a patient. Whilst these are difficult to measure directly, it may be that administrative measures are a better proxy marker than PREMs for these aspects of continuity.

## Conclusions/future work

This review shows a potential emerging relationship between patient reported continuity and health outcomes. However, the evidence for this association is currently weaker than that demonstrated in previous reviews of administrative measures of continuity.

If continuity is to be measured and improved, as is being proposed in some health systems [44], these findings have potential implications as to what type of measure we should use. Measurement of health system performance often drives change [45]. Health systems may respond to calls to improve continuity differently, depending on how continuity is measured. Continuity PREMs are important and patient experienced continuity should be a goal in its own right. However, it is the fact that continuity is linked to multiple positive health care and health system outcomes that is often given as the reason for pursing it as a goal [8, 44, 46]. Whilst this review shows there is emerging evidence of a link, it is not as strong as that found in studies of administrative measures. If, as has been shown in other work, PREMS and administrative measures are looking at different things [23, 24], we need to choose our measures of continuity carefully.

Larger studies are required to confirm the emerging link between patient experienced continuity and outcomes shown in this paper. Future studies, where possible, should collect both administrative and patient reported measures of continuity and seek to understand the relative importance of the three different aspects of continuity (relational, informational, managerial). The relationship between patient experienced continuity and outcomes is likely to vary between different groups and future work should examine differential effects in different patient populations There are now several validated measures of patient experienced continuity [17, 20, 21, 42]. Whilst there may be an argument more should be developed, the use of a standardised questionnaire (such as the Nijmegen questionnaire) where possible, would enable closer comparison between patient experiences in different healthcare settings.

### Electronic supplementary material

Below is the link to the electronic supplementary material.

Supplementary Material 1

Supplementary Material 2

## Data Availability

The datasets used and/or analysed during the current study available from the corresponding author on reasonable request.
